# Strain alignment: toward assessing mechanical plausibility of predicted displacement fields

**DOI:** 10.1007/s11548-026-03729-6

**Published:** 2026-07-02

**Authors:** Bianca Güttner, Micha Pfeiffer, Stefanie Speidel

**Affiliations:** 1 Department of Translational Surgical Oncology, National Center for Tumor Diseases (NCT), NCT/UCC Dresden, a partnership between DKFZ, Faculty of Medicine and University Hospital Carl Gustav Carus, TUD Dresden University of Technology, and Helmholtz-Zentrum Dresden-Rossendorf (HZDR), Dresden, Germany; 2 The Centre for Tactile Internet with Human-in-the-Loop (CeTI), TUD Dresden University of Technology, Dresden, Germany

**Keywords:** Evaluation, Non-rigid registration, Soft tissue, Organ deformation

## Abstract

****Purpose**:**

Commonly used accuracy metrics for non-rigid registration such as target registration error (TRE) do not assess the mechanical plausibility of predicted deformations. We introduce a metric to quantify local coherence of deformation and complement existing measures of physical validity.

****Methods**:**

We propose the strain alignment metric, which evaluates local directional consistency of deformation by analyzing the principal directions and magnitudes of the Green strain tensor. The metric combines three components: (i) a sign term penalizing local flips between compression and extension, (ii) a magnitude term capturing relative variation in principal strain magnitude within a neighborhood and (iii) an angular term measuring non-directional alignment of principal strain directions. The metric is computed locally and summarized over the mesh. We compare it to the Jacobian determinant and strain norm on synthetic liver deformations registered using a deep learning-based method and a fast biomechanical FEM approach.

****Results**:**

While both methods achieve comparable TRE, the proposed metric captures substantial differences in deformation coherence on the sample level that the Jacobian determinant and strain norm only show upon single mesh inspection. It is able to clearly differentiate the largely coherent deformation despite volume changes of the biomechanical method from the local inconsistencies of the learning-based method, including alternating compression and extension patterns.

****Conclusion**:**

Strain alignment provides complementary, interpretable information about the mechanical plausibility of predicted displacement fields. It enables quantitative assessment of local deformation coherence without requiring material assumptions and further highlights limitations of accuracy-only evaluation in non-rigid registration.

**Supplementary Information:**

The online version contains supplementary material available at https://doi.org/10.1007/s11548-026-03729-6.

## Introduction

Computer-assisted navigation requires the registration of preoperative volumetric information to the partially visible, deformed and often noisy surface of the tissue of interest. Under unknown boundary conditions, deep learning-based methods and fast biomechanical methods compete for speed and accuracy. The latter is most commonly reported in terms of a target registration error (TRE) [1–3] or by surrogates such as surface/fiducial errors [4, 5] or reprojection errors [6]. Algorithm evaluation remains challenging because available phantom and in vivo datasets provide ground truth (GT) only at sparse landmarks and lack material information.

***Displacement field quality*** More recently, registration analysis has shifted toward properties of the displacement field. Balakrishnan et al. examined displacement maps from a differential geometry perspective using $\operatorname {det}J_\phi$ which is more closely related to the displacement gradient [7]. From a biomechanical standpoint, Heiselman et al. demonstrated that a low TRE does not guarantee physical plausibility of the predicted displacement field [8], introducing the Jacobian determinant *J* and the strain norm ||ε|| as quantitative metrics (with *J* here equivalent to |*J*| in [8]).

Both metrics can be applied directly to network predictions, as they require no material model, and are computed per element of a discretized mesh. *J* describes local volume changes in the material, with J>1 indicating extension, J<1 signifying compression and J≈1 for nearly incompressible soft tissue. The strain norm reflects the total amount of deformation of an element; however, the value is difficult to interpret without an expected reference value. The directional meaning of the individual strain tensor components is lost in the norm. As a consequence, Heiselman et al. mainly use it to indicate the locality of deformation in a mesh rather than for summary statistics.

*J* remains interpretable without a dense GT displacement field, but relevant spatial relationships are lost when summarized over a mesh. Moreover, it does not capture local coherence of deformation direction.

A measure is needed that alleviates these disadvantages. Like *J*, it should directly be applicable to network predictions and interpretable without dense GT. To this end, we propose the strain alignment metric γ.

## Metric definition

***Foundations*** We adapt the mean alignment metric $\bar{\gamma }$ from the analysis of swarming behavior in a discrete lattice-gas cellular automaton (LGCA) model by Bouré et al. [9] to the continuous-domain deformation problem. It requires a vector field with a meaningful sign change and values centered around 0.

As a basis for the field we use the Green strain $\textbf{E} = \frac{1}{2} (\textbf{F}^T \textbf{F} - \textbf{I}) = \frac{1}{2} ( \nabla \textbf{u}^T + \nabla \textbf{u} + \nabla \textbf{u}^T \nabla \textbf{u})$ with deformation gradient $\textbf{F}$, identity matrix $\textbf{I}$ and displacement between undeformed and deformed state $\textbf{u}$, where variables in bold indicate vectors and tensors. It is invariant to rigid body motion with $\textbf{E}=0$ for purely rigid displacements (no deformation); nonzero values therefore capture only the soft tissue deformation. It shows a sign flip between compression ($E_{ij}<0$) and extension ($E_{ij}>0$) with a smooth transition between the domains. In order to assess the deformation direction and decouple the values from the specific spatial discretization, we use the principal strains (eigenvalues $E_i, \ i=1...3$ of $\textbf{E}$) and their associated normalized eigenvectors $\textbf{v}_{Ei}$. $\textbf{v}_{Ei}$ constitute the material axes along which the material only experiences normal strain.

***Strain alignment*** We reduce the complexity by focusing on the largest principal value (by absolute value) $E_1$, which presents the largest influence on the material. Its values are interpretable, for example, $E_1=0.2$ roughly corresponds to 20% stretch (strictly: 18%) along $\textbf{v}_{Ei}$, and can be related to material curves. With this, we conceptually construct an undirected vector field with angular directions $\textbf{v}_{E1}$ and magnitudes $E_1$ that indicates the ”main direction of deformation” for each mesh element.

We define the set of mesh elements $\mathcal {T}$ with the number of elements $T = \operatorname {card}(\mathcal {T})$, the element centroids $\textbf{r}: \mathcal {T} \rightarrow \mathbb {R}^3$ and a local neighborhood $\mathcal {N}$ of each element *t* constrained by a radius *d* around its centroid:

$$\begin{aligned} \mathcal {N}^{(t)} = \{ n \in \mathcal {T} \setminus \{t\} | \, || \textbf{r}(n) - \textbf{r}(t)||_2 < d\}, \end{aligned}$$

where $||\cdot ||_2$ denotes the L2 matrix norm. $\mathcal {N}^{(t)}$ is defined on the undeformed mesh.

The maximum principal values of the neighboring elements are condensed into the director field magnitude $D^{(t)} = \sum _{n \in \mathcal {N}^{(t)}} E_1^{(n)}$. With these components we adapt the metric as:with $\operatorname {sgn}(.)$ as the sign function, |.| the absolute value, $\varphi (\textbf{a}, \textbf{b}) = \operatorname {arccos}\left( \frac{\textbf{a} \cdot \textbf{b}}{||\textbf{a}||_2 ||\textbf{b}||_2} \right)$ the angle between vectors, · the dot product. In contrast to the original LGCA application, we recommend using the more robust median instead of the mean for mesh-wide summarization. To avoid confusion and consistent with its derivation from the Green strain, we call the metric *strain alignment*.

γ is constructed such that the sign term punishes sign flips between compression and extension in the neighborhood, which are expected to occur very gradually in real soft bodies. The magnitude term penalizes large local changes in the maximum principal value, normalized to be $\gamma ^{(t)} = \gamma _s^{(t)} = \gamma _m^{(t)} = \gamma _a^{(t)} = 1$ for a constant field or a smooth linear increase (examples in the Online Resource, Fig. S1).

The angle term is designed to allow directions antiparallel to the local neighborhood to account for the undirectedness of the material axes. The sum over all neighbors is normalized to a maximum of 1. The minimum angular alignment $\gamma _a^{(t)} = 0$ is reached for an angle of $90\,^{\circ }$ to all surrounding vectors (more details in the Online Resource, section S1.1).

## Experiments

**Fig. 1:**
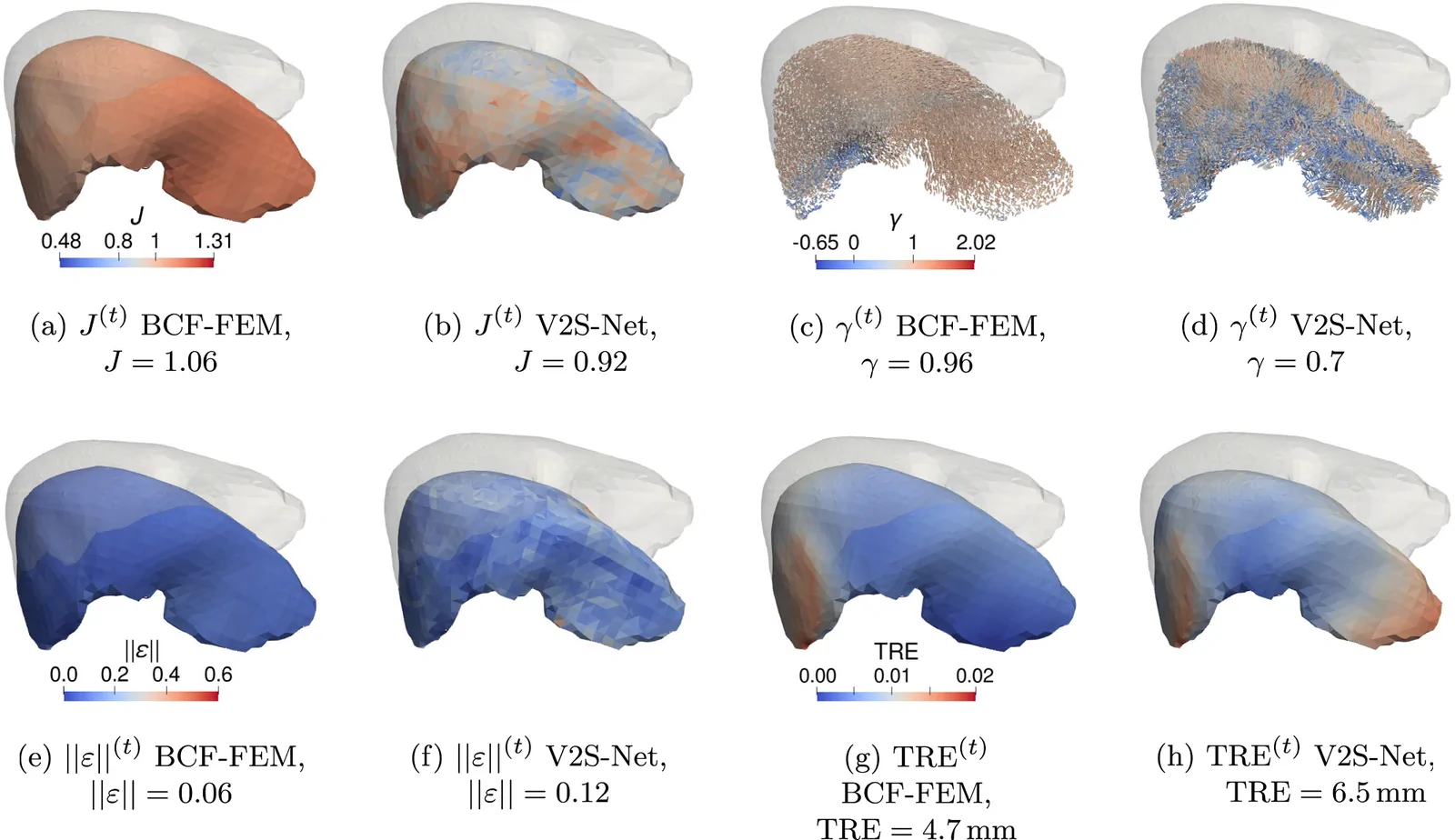
Example mesh (deformed, in color) with spatial distribution of the metrics. The undeformed mesh is shown transparently. The glyph for $\gamma ^{(t)}$ is oriented along the axis of the largest principal strain $\textbf{v}_{E1}$. $\gamma ^{(t)}$ reliably detects shifts in the dominant deformation direction that cause locally extensive and compressive areas roughly visible through $J^{(t)}$

**Fig. 2:**
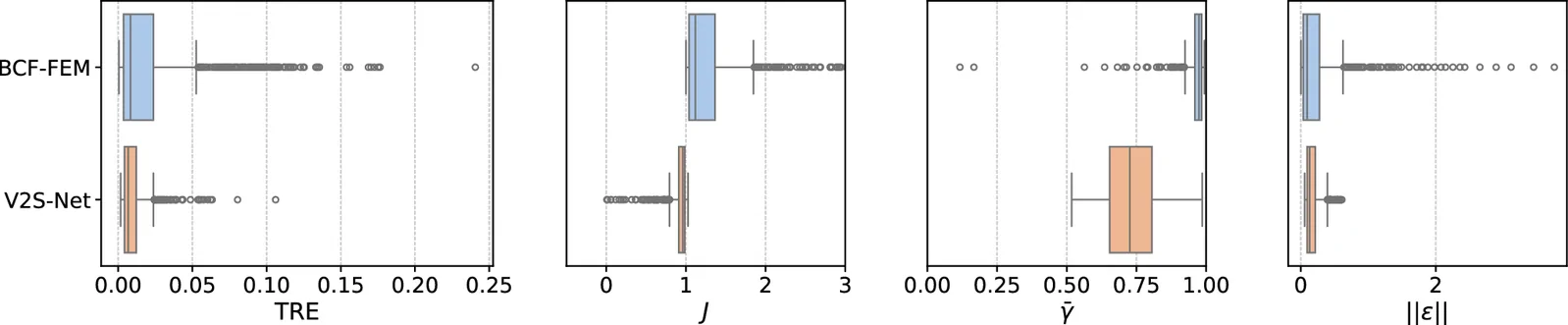
Analysis of BCF-FEM (blue) and V2S-Net (orange) inference results. Left to right: Target registration error, Jacobian determinant, strain alignment, strain norm. A data point corresponds to the median over the mesh elements. Boxplot measures: line: median, box: quartiles, whiskers: 1.5 inter-quartile range. Some outliers in *J* for BCF-FEM have been cropped for better visibility, max. value 6.307. V2S-Net outperforms BCF-FEM with respect to TRE. Both methods show large deviations from J=1. $\tilde{\gamma }$ assumes lower values with higher variation for V2S-Net, which hints at undesirable local deformation patterns

**Fig. 3:**
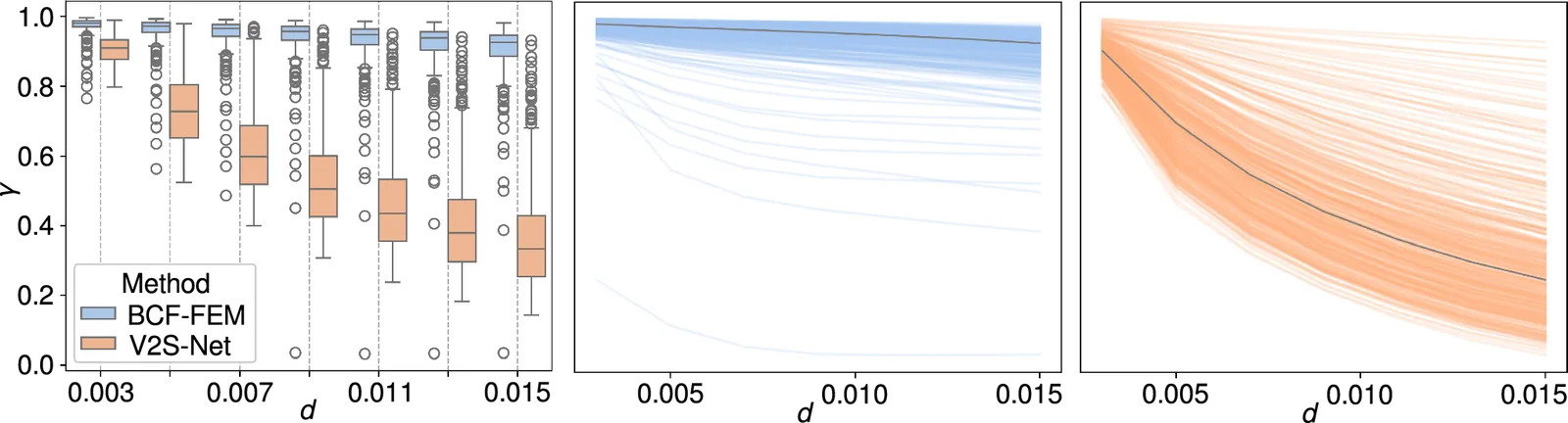
Effect of different *d* on γ and $\gamma ^{(t)}$. A line corresponds to a sample, the gray line to the dataset median. Higher *d* separate the methods more prominently. The sample ranking is robust to the choice

**Fig. 4:**
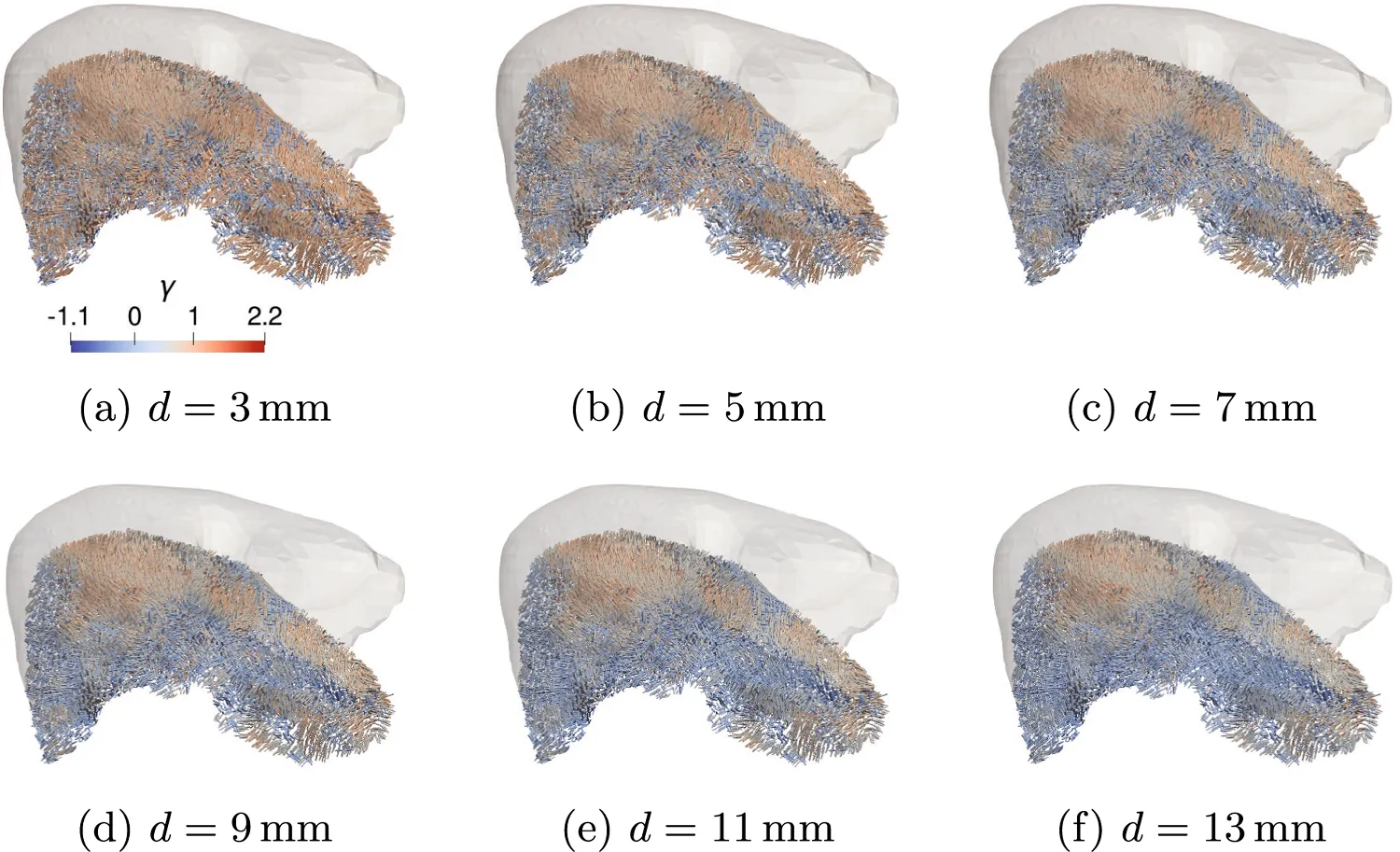
Example mesh (deformed, in color) with spatial distribution of $\gamma ^{(t)}$ for different *d* on inference results of V2S-Net. The undeformed mesh is shown transparently. Glyphs are oriented along the axis of $\textbf{v}_{E1}$. At smaller *d*, $\gamma ^{(t)}$ reflects local misalignments more clearly

We demonstrate the applicability of γ by analyzing inference results of a deep learning-based method (V2S-Net [2]) and a biomechanical method (BCF-FEM [3]) on a synthetic registration dataset and compare it to the established Jacobian determinant and strain norm. 628 liver meshes were reconstructed from the AMOS dataset [10], deformed synthetically using a finite element formulation with a neo-Hookean material and reduced to a partial surface as the registration target. During the evaluation *d* was set to 5 mm, corresponding to the minimum mesh resolution of the dataset.

***Spatial distribution*** Fig. 1 shows the spatial distribution of all metrics for one sample seen from the anterior. The lower right lobe was subjected to fixed boundary conditions, with the partial target surface located on the lower left lobe. Highlighting the unrealistic enlargement of the left lobe by BCF-FEM, $J^{(t)}$ exposes the increase in local volume, likely a consequence of the linear material model unsuitable for this amount of deformation. Despite this artifact, $\gamma ^{(t)}$ shows a smooth glyph field, indicating alignment in the deformation axes and a subsequently high median value as expected for a biomechanical method. For V2S-Net, $J^{(t)}$ indicates unrealistic local extension and compression due to an incoherently oriented deformation field as illustrated by $\gamma ^{(t)}$. It can be explained by the network moving points independently from each other, which can cause arbitrary changes to the shapes of neighboring mesh elements and consequently qualitatively different deformations. Low $\gamma ^{(t)}$ can be found where the direction changes, surrounding patches with more coherent movement that also show local agreement in $J^{(t)}$ and leading to a γ for the mesh that is clearly distinct from perfect alignment at 1. This is spatially not correlated to the TRE. $||\varepsilon ||^{(t)}$ shows similar spatial patterns to $J^{(t)}$, likely because they are both related to $\textbf{F}$, but besides the general limits for strain magnitudes related to tissue damage its value remains uninformative without reference values from a dense ground truth.

***Dataset distribution*** Fig. 2 shows the summarized results for the full dataset. V2S-Net achieves a lower TRE than BCF-FEM and Jacobian values closer to 1 despite the spatial inconsistencies elucidated in Fig. 1. Poor values within a mesh can cancel each other out. Spurious extension due to the linear material model at the base of BCF-FEM remains visible. ||ε|| shows comparable values between the methods with a higher variation for BCF-FEM and overall more deformation for V2S-Net. It does not indicate which method performs better, i.e., whether the larger median deformation for V2S-Net reflects strong dataset deformations or method-induced errors. In contrast, γ allows a clear distinction between the methods with respect to spatial deformation patterns. The qualitative differences shown in Fig. 1 successfully translate into the summarized value.

For BCF-FEM, values of γ predominantly close to 1 indicate that except for some outliers, probably failed cases, the material moves coherently. The strain alignment shows lower values for V2S-Net, with a much larger variation across samples.

Since the strain alignment does not allow any conclusions about the strength of deformation, a combination of γ and the Jacobian determinant provides the most information about the properties of a predicted displacement field.

## Discussion and conclusion

The proposed strain alignment metric provides complementary information but requires careful interpretation. The magnitude term could be capped at 1 to ensure a strict upper bound, and alternative characterizations of local gradient structure may be explored. By definition, the metric depends on the choice of *d*. It must be large enough relative to mesh resolution to define a meaningful neighborhood, yet excessive values introduce smoothing effects. The influence of *d* on γ is shown in Fig. 3 and 4.

Further limitations arise from the formulation. Large and small strains are weighted equally, so noise near zero may affect the metric disproportionately. The reduction to the maximum principal strain can also introduce ambiguity: When $E_1$ and $E_2$ are similar, variations in eigenvector orientation may not reliably characterize the underlying deformation. If the registration method operates on an alternative representation (e.g., voxels), interpolation onto the mesh may introduce artifacts since the metric is sensitive to changes in the geometry of neighboring mesh elements. In addition, it can exhibit multimodal distributions across the mesh, motivating work toward more informative summary statistics.

Overall, *J* and γ complement each other in dataset-level analyses, with γ capturing local directional coherence. Unlike stress- or force-based measures, both require no material assumptions and can be applied to any mesh deformed by a predicted displacement field. The key contribution lies in the conceptual shift toward leveraging the principal strain coordinate system to characterize deformation fields in registration evaluation.

Future work will explore alternative reductions of the system to a scalar. A remaining challenge is the hierarchical distribution of metric values across mesh elements and dataset samples.

## Supplementary Information

Below is the link to the electronic supplementary material.

Supplementary file 1 (pdf 203 KB)
