# Supplementary material for: Strain alignment: toward assessing mechanical plausibility of predicted displacement fields
Source: Int J Comput Assist Radiol Surg. 2026 Jul 2;21(7):1689–93. doi: 10.1007/s11548-026-03729-6 (PMC13553697; doi:10.1007/s11548-026-03729-6)
Supplement: Supplementary file 1 [file 11548_2026_3729_MOESM1_ESM.pdf]

Strain Alignment: Towards Assessing Mechanical Plausibility of Predicted Displacement Fields

Bianca Güttner*, Micha Pfeiffer, Stefanie Speidel

Supplementary Information

International Journal of Computer Assisted Radiology and Surgery

S1 Additional Details on Metric Formulation

S1.1 Metric Design Remarks

Not using stretch

When inquiring about the amount of deformation in percent, often the stretch $\mathbf{U} = \left(\mathbf{F}^T\mathbf{F}\right)^{\frac{1}{2}}$ is considered rather than the strain, with eigenvalues $\lambda_i = \sqrt{2E_i + 1}$. However, the distinction between compression and extension is placed at 1 instead of 0 and would need to be centered in order to work with the sign change requirement and to maintain its symmetry.

Domain transfer

In order to transfer the metric from the discrete [1] to the continuous domain and to our problem, we take several steps: The possibility of varying values in $E_1^{(n)}$ (as opposed to unit length of the vectors in the LGCA) makes the magnitude term necessary to capture local deviations. The sum of the neighbourhood principal values is put in relation to the number of neighbours times the central value as a reference.

For the angle term, since we do *not* want angle misalignments to cancel each other out, the angle between the vectors is assessed pairwise instead of summarizing the neighbourhood vectors into the original director field vector $D_c(\mathbf{x})$ first (maintaining the notation from Bouré et al. [1]). Since our vectors are undirected (material axes) rather than directed (LGCA velocity field), we use the squared cosine instead of the cosine such that we obtain perfect alignment for antiparallel vectors. The cosine is implicit in the dot product in the referenced paper.

*bianca.guettner@nct-dresden.de

S1.2 Example Fields

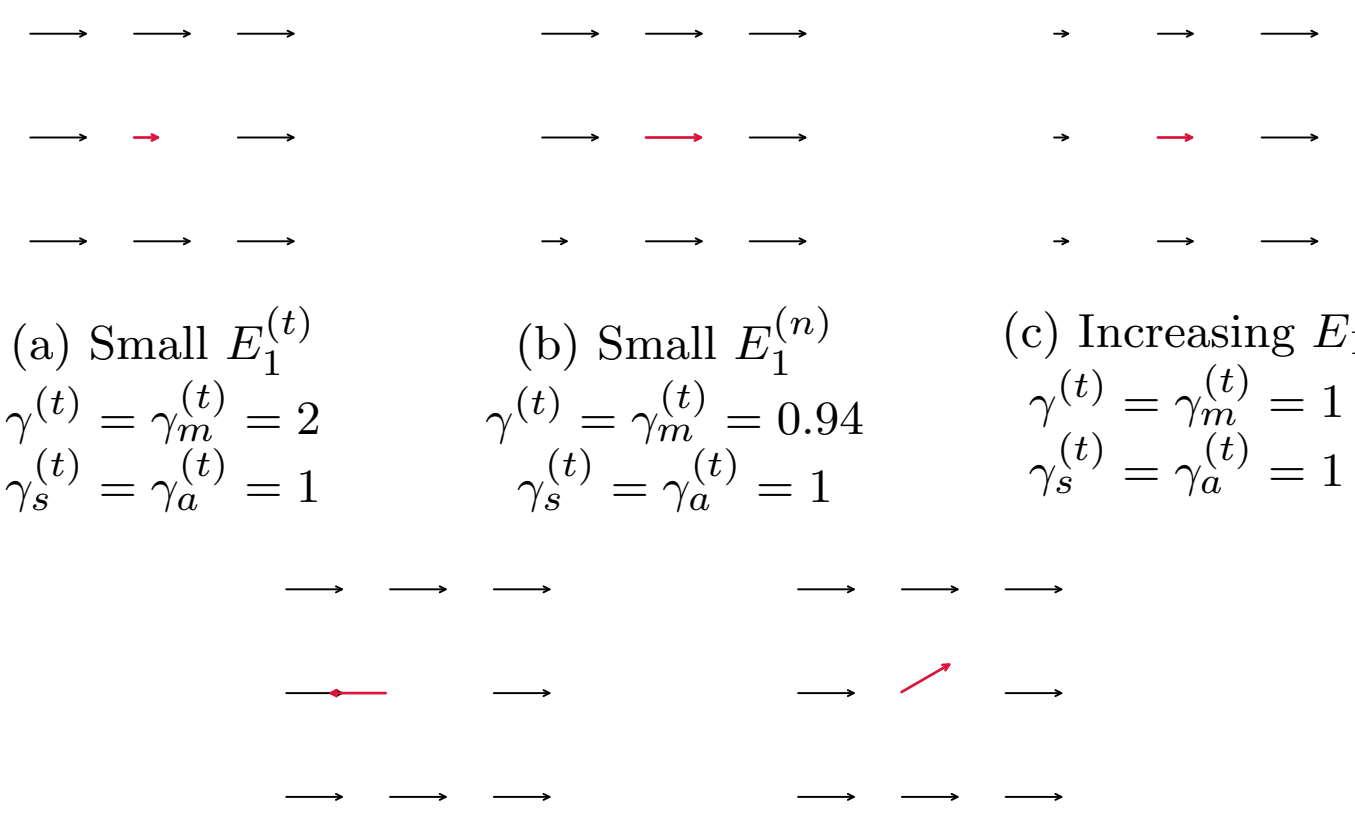

(a) Small $E_1^{(t)}$
$\gamma^{(t)} = \gamma_m^{(t)} = 2$
$\gamma_s^{(t)} = \gamma_a^{(t)} = 1$

(b) Small $E_1^{(n)}$
$\gamma^{(t)} = \gamma_m^{(t)} = 0.94$
$\gamma_s^{(t)} = \gamma_a^{(t)} = 1$

(c) Increasing E_1
$\gamma^{(t)} = \gamma_m^{(t)} = 1$
$\gamma_s^{(t)} = \gamma_a^{(t)} = 1$

(d) $E_1^{(t)}$ sign flip
$\gamma^{(t)} = \gamma_s^{(t)} = -1$
$\gamma_m^{(t)} = \gamma_a^{(t)} = 1$

(e) $\mathbf{v}_{E1}^{(t)}$ angle diff.
$\gamma^{(t)} = \gamma_a^{(t)} = 0.75$
$\gamma_s^{(t)} = \gamma_m^{(t)} = 1$

Fig. S1: Example fields to showcase the metric interpretation. Arrows are oriented along the axis of $\mathbf{v}_{E1}$, scaled and directed by E_1. Red represents the tetrahedron of interest t, black the ones in its neighborhood $n \in \mathcal{N}^{(t)}$. The first line indicates the main influencing term for the respective situation.

References

[1] Bouré, O., Fatès, N., Chevrier, V.: A Robustness Approach to Study Metastable Behaviours in a Lattice-Gas Model of Swarming. In: Cellular Automata and Discrete Complex Systems vol. 8155. Springer, Berlin, Heidelberg (2013). https://doi.org/10.1007/978-3-642-40867-0_6
